# Effects of manipulating the duration and intensity of aerobic training sessions on the physical performance of rats

**DOI:** 10.1371/journal.pone.0183763

**Published:** 2017-08-25

**Authors:** Francisco Teixeira-Coelho, Cletiana Gonçalves Fonseca, Nicolas Henrique Santos Barbosa, Filipe Ferreira Vaz, Letícia Maria de Souza Cordeiro, Cândido Celso Coimbra, Washington Pires, Danusa Dias Soares, Samuel Penna Wanner

**Affiliations:** 1 Exercise Physiology Laboratory, School of Physical Education, Physiotherapy and Occupational Therapy, Universidade Federal de Minas Gerais, Belo Horizonte (MG), Brazil; 2 Teacher Formation Center; Universidade Federal do Recôncavo da Bahia, Amargosa (BA), Brazil; 3 Laboratory of Endocrinology and Metabolism, Institute of Biological Sciences, Universidade Federal de Minas Gerais, Belo Horizonte (MG), Brazil; 4 Department of Physical Education, Institute of Life Sciences, Universidade Federal de Juiz de Fora, Governador Valadares (MG), Brazil; Universidad Europea de Madrid, SPAIN

## Abstract

This study investigated the effects of manipulating the load components of aerobic training sessions on the physical performance of rats. To achieve this purpose, adult male Wistar rats were divided into four groups: an untrained control (CON) group and training groups with a predominant overload in intensity (INT) or duration (DUR) or alternating and similar overloads in intensity and duration (ID). Prior to, during, and after 8 weeks of the control or training protocols, the performance of the rats (evaluated by their workload) was determined during fatiguing, incremental-speed treadmill running. Two additional incremental running tests were performed prior to and at the end of the protocols to measure the peak rate of oxygen consumption (VO_2peak_). As expected, the rats in the trained groups exhibited increased performance, whereas the untrained rats showed stable performance throughout the 8 weeks. Notably, the performance gain exhibited by the DUR rats reached a plateau after the 4^th^ week. This plateau was not present in the INT or ID rats, which exhibited increased performance at the end of training protocol compared with the DUR rats. None of the training protocols changed the VO_2peak_ values; however, these values were attained at faster speeds, which indicated increased running economy. In conclusion, our findings demonstrate that the training protocols improved the physical performance of rats, likely resulting from enhanced running economy. Furthermore, compared with overload in duration, overload in the intensity of training sessions was more effective at inducing performance improvements across the 8 weeks of the study.

## Introduction

Training is a complex process of overload induction to disturb body homeostasis and produce acute fatigue, which leads to physical performance improvement if the training sessions are repeatedly performed with appropriate recovery periods [1]. Thus, fatigue and the resulting adaptations induced by exercise sessions depend on the applied training loads. In general, these loads are characterized by the intensity, duration and frequency of exercise sessions [2], with the combination of the latter two factors determining the training volume (i.e., in the case of endurance training, training volume can be expressed as the distance traveled per week or month). It is widely accepted that the optimization of these load components produces adaptive responses that increase physical performance [2].

Distinct manipulation of the load components (e.g., intensity and volume) differentially modulates molecular signaling in skeletal muscle [3] and thus may differentially affect physical performance. However, there is no consensus on how training programs should be structured (the ideal moment for applying overload in these load components or which load component should be predominantly manipulated) to optimize performance gains [3]. In particular, the efficacy of low-intensity long-duration training sessions and the efficacy of increasing training volumes in improving performance remain controversial [4–6].

A seminal study by Helgerud et al. [7] compared different training protocols and observed that performing high-intensity interval training was significantly more effective than performing the same total work at or below the lactate threshold for improving the maximum rate of oxygen consumption (VO_2max_) in healthy university students. Similarly, a recent meta-analysis [8] indicated that high-intensity interval training provides greater VO_2max_ gains than moderate-intensity continuous training in individuals with cardiometabolic diseases. Based on these data, we hypothesized that a predominant overload in the intensity of aerobic training sessions would produce more pronounced adaptations than a predominant overload in the duration, thereby resulting in increased VO_2max_ and physical performance improvement. Therefore, our first aim was to investigate the effects of manipulating the intensity and duration of aerobic training sessions on the VO_2max_ and physical performance of rats subjected to incremental exercise.

VO_2max_ as well as other physiological parameters, such as running economy, are important for determining endurance running performance [9, 10]. In highly trained and experienced distance runners with a similar VO_2max_, running economy accounts for a large amount of the variation observed in performance in a 10 km race [11]. Regarding the changes in running economy induced by different training protocols, Franch et al. [12] observed that recreational runners had improved economy and performance with exhaustive distance training or long-interval running, whereas short-interval running was less efficient. Considering these findings, we hypothesized that predominant overload on duration would produce greater improvements in running economy. Thus, we also investigated the effects of manipulating the intensity and duration of aerobic training sessions on the running economy of rats.

To adequately investigate the effectiveness of protocols with predominant overload in intensity or duration on performance improvement, we selected untrained rats that began training with the same load and were subjected to the same overload in their training volume. The present experiments were conducted with laboratory rodents, which enabled the standardization of age, physical exercise experience and intrinsic endurance at training initiation as well as their level of physical activity and diet outside the training environment. Unlike other studies, we did not simply compare protocols with different intensities using rats that had already begun training at different loads [13] or compare high-volume against high-intensity protocols [14]; the present study focused on the divergent effects of distinct overload progressions applied to rats that began training with a similar load. Therefore, we equalized the number of weekly sessions, the distance covered in each session and, consequently, the training volumes among the trained groups, and then overload was predominantly applied to the intensity or duration of the exercise sessions. In addition, these protocols (i.e., predominant overload in intensity or duration) were also compared with a traditional protocol, in which the overload was applied by alternately increasing the intensity and duration of the training sessions.

Additionally, heterogenic lineages of laboratory rats show large variability in their intrinsic endurance capacities, which are associated with differences in their cardiac functioning [15] and central nervous system [16]. Because previous studies in humans have shown that initial fitness levels determine the magnitude of physiological adaptations to training [2], we hypothesized that rats with lower intrinsic endurance would present greater performance gains. Moreover, the degree and rate at which physiological systems and intense exercise performance change in the short term, at least in well-conditioned subjects, appear to be affected more by high-intensity training than by high-volume training [17]. Thus, it is likely that rats with higher intrinsic endurance (i.e., those with lower adaptive potential in response to training) would need more intense stimuli than rats with lower intrinsic endurance. We therefore expected that a predominant overload in intensity would be required to maximize the ergogenic effects induced by training in rats with higher intrinsic aerobic capacity. Therefore, the effects of the three aerobic trainings on performance in rats with different intrinsic aerobic capacities were also evaluated.

## Materials and methods

### Animals

All experimental procedures were approved by the Ethics Commission of the Universidade Federal de Minas Gerais for the Care and Use of Laboratory Animals (protocol 64/2014) and were conducted in accordance with the regulations provided by the Brazilian National Council for the Control of Animal Experimentation. Adult male Wistar rats weighing 220–250 g were housed in collective cages under controlled temperature (24 ± 1°C) and light conditions (lights on from 0500 until 1900 h) with water and rat chow provided *ad libitum*.

### Familiarization with running on a treadmill

The rats were initially familiarized with running on a treadmill designed for small animals (Gaustec Magnetismo; Nova Lima, MG, Brazil) during 5 consecutive days; the duration and speed of each familiarization session are described in Table 1. This 5-day protocol was used to teach the rats the direction to run, i.e., away from the electrical stimulus grid placed at the end of the treadmill belt. Following the familiarization sessions, all rats exhibited a steady running pattern, with minimal exposure to electrical stimuli (0.5 mA).

**Table 1:** Five-day familiarization protocol with running on a treadmill.

1^st^ day	2^nd^ day	3^rd^, 4^th^ and 5^th^ days
5 min (rest)	5 min (rest)	5 min (rest)
1 min (10 m/min)	1 min (12 m/min)	5 min (15 m/min)
1 min (12 m/min)	5 min (15 m/min)	
5 min (15 m/min)		

The familiarization sessions were performed with a treadmill inclination of 5° and in a room with controlled temperature conditions (23 ± 1°C), which were similar across all training and incremental exercise sessions.

### Training protocols

The 84 rats were allocated to four groups (n = 21 each): an untrained control (CON) group, a training group with predominant overload in intensity (INT), a training group with predominant overload in duration (DUR) and a training group with alternating and similar overloads in intensity and duration (ID). A homogeneous distribution of rats among the experimental groups was guaranteed by matching the mean values and coefficient of variations of the intrinsic aerobic capacity, which were evaluated by the workload performed during the first test.

**Fig 1:**
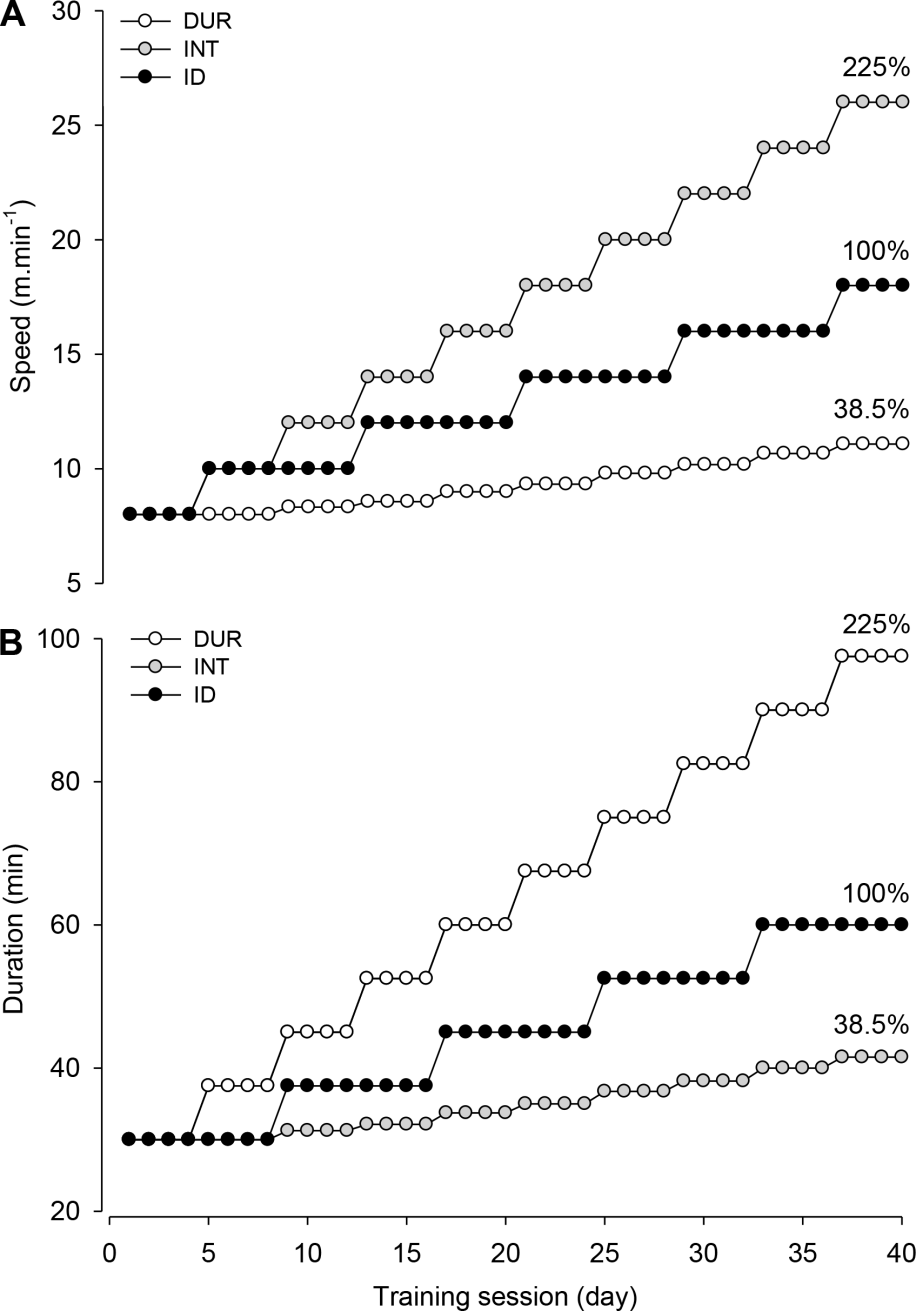
Overload during the training protocols. Description of the increases in treadmill speed (intensity; panel A) and running time (duration; panel B) during the 40 training sessions in each of the three trained groups. Each circle represents one training session.

The training protocols consisted of running sessions on a treadmill across 8 weeks, with 5 weekly sessions always performed at the same time of day. The three protocols used in this study are described in detail in the Supporting Information section of the manuscript (S1–S3 Tables) and were adapted from a protocol proposed by Santiago et al. [18]. At the last session of the aerobic training protocols, the increase in speed relative to the first session corresponded to 225% (INT), 125% (ID) and 38.5% (DUR; Fig 1A), whereas the increase in duration corresponded to 38.5%, 100% and 225% in the INT, ID and DUR groups, respectively (Fig 1B). Despite the different overload protocols, the total distance covered by the rats during each training session was the same among the three trained groups. To ensure similar handling and exposure to the treadmill setup, the CON rats were subjected to 2 min of treadmill running 5 days per week, with the same running speed as the ID rats.

The rats that lost their ability to exhibit a steady run on the Gaustec treadmill and were subsequently exposed to frequent, light electrical stimuli as aerobic training progressed were transferred to run on another treadmill setup (Panlab/Harvard Apparatus). This strategy was successful because it avoided excluding rats from the training protocol without interfering with performance gains.

### Incremental-speed treadmill running for assessing the workload performed by the rats

All 84 rats were subjected to incremental-speed exercises to assess the time to fatigue and workload before (first test) and at the ends of the 4^th^ (second test) and 8^th^ weeks (third test). All these incremental-speed exercises were performed at least 24 h after the last familiarization session (first test) and the last training session for the week (second and third tests). During the incremental exercises, the rats began running at a speed of 10 m.min^-1^, followed by speed increments of 1 m.min^-1^ every 3 min until they were fatigued [15]. Volitional fatigue was defined as the point at which the animals were no longer able to maintain their pace with the treadmill, even when exposed to light electrical stimulation for 10 s [19, 20].

### Incremental-speed treadmill running for assessing the peak rate of oxygen consumption (VO_2peak_) and running economy

Thirty-three of the 84 rats (8 rats in each training group and 9 rats in the control group) were subjected to additional incremental exercises on the Panlab treadmill running setup (Panlab/Harvard Apparatus, Cornella, Spain) before and after the training protocol (first and second VO_2_ testing, respectively). The aim of these two incremental exercises was to assess running economy and the VO_2max_ attained by the rats.

This subgroup of 33 rats was therefore subjected to five incremental exercises: two before, one during and two after the control or training protocols. Whenever two exercises were performed in sequence (i.e., before and after), the first was performed on the Gaustec treadmill to assess workload (at least 24 h after the last familiarization session or the last training session for the eight week), whereas the second was performed on the Panlab treadmill to assess VO_2max_, running economy and workload (48 h following the first exercise). Despite the fact that two different setups were used, the workload performed by the same rat during exercise in the two treadmills was significantly correlated either before or after training (*p* < 0.001 for both, Pearson’s correlation).

### Euthanasia

After the last experimental trial, the rats were euthanized through an intraperitoneal injection of a lethal dose of anesthetic (ketamine 240 mg/kg and xylazine 30.5 mg/kg).

### Measurements

The rats were weighed daily; changes in body mass were used as an index of health and hydration status during the training protocols. The time to fatigue (in min) corresponded to the time elapsed between the start and end of the fatiguing incremental exercises. The VO_2_, expressed in size-adjusted units (mL.kg^-0.75^.min^-1^), was continuously measured via open-flow indirect calorimetry (Panlab/Harvard Apparatus). The VO_2_ data were transferred to a computerized system (Metabolism, Panlab/Harvard Apparatus), which was calibrated weekly using a known mixture of gases to ensure the reliability of the evaluated data. In most of the rats evaluated, we could not observe a clear VO_2_ plateau with increased treadmill speed. Thus, the VO_2peak_ was determined instead of the VO_2max_. The VO_2peak_ consisted of the highest VO_2_ value measured, usually at fatigue. Because the rats’ body mass increased by ~67% across the 8 weeks, the VO_2_ data were expressed in relation to body mass raised to the power of 0.75 [21].

The temperature inside the treadmill chamber was measured every min during the incremental exercises (except for the experiments with VO_2_ measurements) via a thermocouple attached with impermeable adhesive tape to the ceiling of the acrylic chamber containing the treadmill belt.

### Calculations

The maximum speed (S_max_; m.min^-1^) attained was calculated according to the following equation: S_max_ = S + (t/180), where S = speed in the last completed stage in m.min^-1^, and t = time spent in the incomplete stage in seconds [22]. The workload (J) was calculated as follows: workload = m⋅g⋅s⋅sinθ⋅t, where m = body mass in kg, g = gravity force (9.8 m.s^-2^), s = speed in m.min^-1^, θ = inclination of the treadmill (5°), and t = time spent in each stage in min [23]. Workload values were calculated for each stage of the incremental exercise, including the incomplete stage and were then summed; the value obtained after the summation corresponded to the exercise workload.

Two intensity indices of the training sessions were calculated: the percentages of S_max_ (%S_max_) and VO_2peak_ (%VO_2peak_). The %S_max_ was calculated by dividing the treadmill speed in the beginning (first session), middle (21^st^ session) and end (40^th^ session) of the training protocol by the S_max_ attained during the first, second and third tests, respectively; this value was then multiplied by 100. To estimate the %VO_2peak_ associated with physical exertion during the last training session, we verified the existence of a linear correlation between VO_2_ and treadmill speed during the incremental exercise for each rat evaluated; the data regarding VO_2_ and speed at each stage of the exercise were included in this analysis. Because these correlations were significant (*p* was always less than 0.05 following Pearson’s correlation), the equation expressing the linear regression between the two parameters was calculated for each rat, and the %VO_2peak_ corresponding to a given treadmill run was then estimated.

Gross oxygen cost of running, which is inversely associated with running economy, was calculated by dividing the VO_2_ (mL⋅kg^-0.75^⋅min^-1^) by the running speed (m.min^-1^), and the resulting value was expressed in mL⋅kg^-0.75^⋅m^-1^ [24]. Although the gross oxygen cost of running is commonly determined during constant-speed exercises, we calculated this parameter in the last min of the last 3-min stage that was completed during the incremental exercise, since Wisloff et al. [25] reported that VO_2_ leveled off in running rats ∼3 min after the stage was changed.

### Statistical analysis

Shapiro-Wilk and Levene’s tests were used to assess the normality and homoscedasticity of the data, respectively. Because all data presented a normal distribution, they were expressed as the means ± SEM. The body mass, workload, VO_2peak_, running economy and %S_max_ were compared between groups and moments (before, during, and after the 8-week period) using two-way analyses of variance (ANOVAs), with repeated measures applied only for the moment factor. The VO_2_ curves were compared between the running speeds and moments using a two-way ANOVA, with repeated measures applied for both factors. The changes in the workload performed or the VO_2peak_ attained between the first and third tests were compared between the experimental groups and the intrinsic aerobic capacity via a two-way ANOVA. The intensity of the last training session, expressed as the %VO_2peak_, was compared between the groups using a one-way ANOVA. When applicable, these ANOVAs were followed by Tukey’s *post hoc* tests to identify differences between pairs of means. The association between workload and VO_2peak_ during incremental exercises was assessed using Pearson’s correlation coefficient. The significance level was set at α < 0.05.

## Results

The application of the overload principle was evident in the three training protocols; both the duration and intensity (running speed) of the training sessions were successfully increased across the 8-week period (Fig 1A and 1B). Regarding the duration of the training sessions, the DUR group ran for 97.5 min during the last session, which corresponded to a 225% increase relative to the duration of the first session. As expected, intergroup differences were also identified; for example, the rats in the DUR group exhibited 487% and 125% higher increases in duration across the 8 weeks than those exhibited by the rats in the INT and ID groups, respectively.

**Fig 2:**
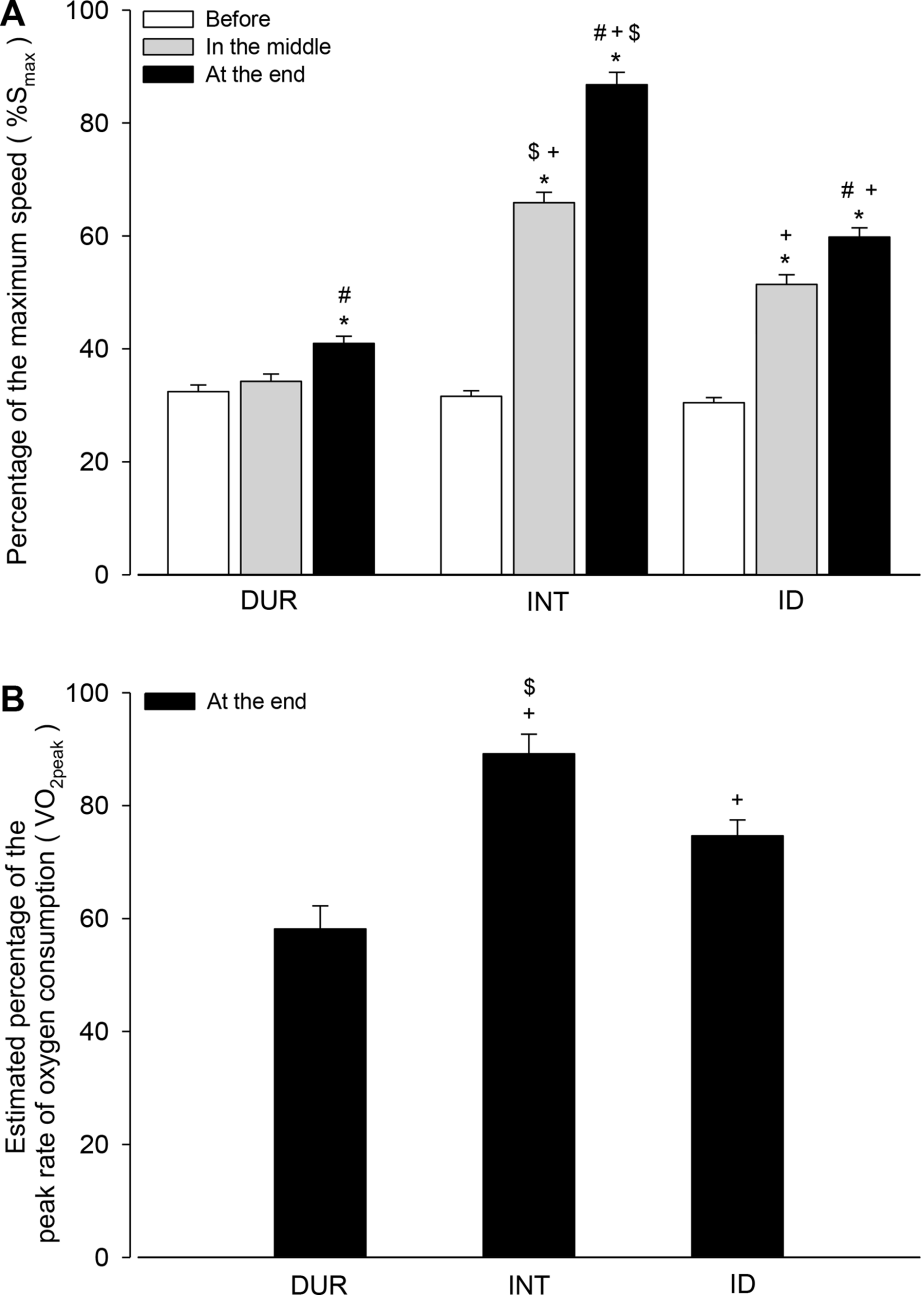
Intensity of the exercise sessions during the three training protocols. Panel A indicates the percentage of the maximum speed at which the rats were running at three different moments: before, in the middle of (beginning of the fifth week) and at the end of the training protocol. Two-way ANOVA yielded the following results: group effect (F = 133.556, *p* < 0.001), moment effect (F = 463.267, *p* < 0.001) and interaction between the two factors (F = 91.745, *p* < 0.001; n = 21 rats per training group). Panel B indicates the estimated percentage of the peak rate of oxygen consumption at which the rats were running during the last session of the training protocol. One-way ANOVA yielded the following result: group effect (F = 19.798, *p* < 0.001; n = 8 rats per training group). * Significantly different relative to the beginning of training within an experimental group (*p* < 0.001). # Significantly different relative to the middle of training within an experimental group (*p* < 0.001). + Significantly different relative to the DUR group in the same test (*p* < 0.001). $ Significantly different relative to the ID group in the same test (*p* < 0.001).

Regarding the intensity of the training sessions, the three trained groups initiated the protocol running at a similar %S_max_ (DUR: 32.4 ± 1.2% vs. INT: 31.6 ± 1.0% vs. ID: 30.5 ± 0.9%; Fig 2A). However, both the INT and ID groups ran at a higher %S_max_ relative to the DUR group in the middle and at the end of the training (Fig 2A). Indeed, the DUR rats showed only a slightly higher %S_max_ at the end than at the beginning of the training (41.0 ± 1.3% vs. 32.4 ± 1.2%). In addition, in the middle and at the end of the training period, the INT group ran at a higher %S_max_ relative to the ID group (middle: 65.9 ± 1.9% vs. 51.4 ± 1.7%, and end: 86.8 ± 2.2% vs. 59.8 ± 1.7%). Similarly, the different running speeds between the trained groups also represented a different estimated %VO_2peak_ in the last training session (Fig 2B). The INT rats ran at increased intensities (89 ± 3% VO_2peak_) compared with the rats in the other groups, and the ID rats ran at increased intensities compared with the DUR rats (75 ± 3% vs. 58 ± 4% VO_2peak_).

**Fig 3:**
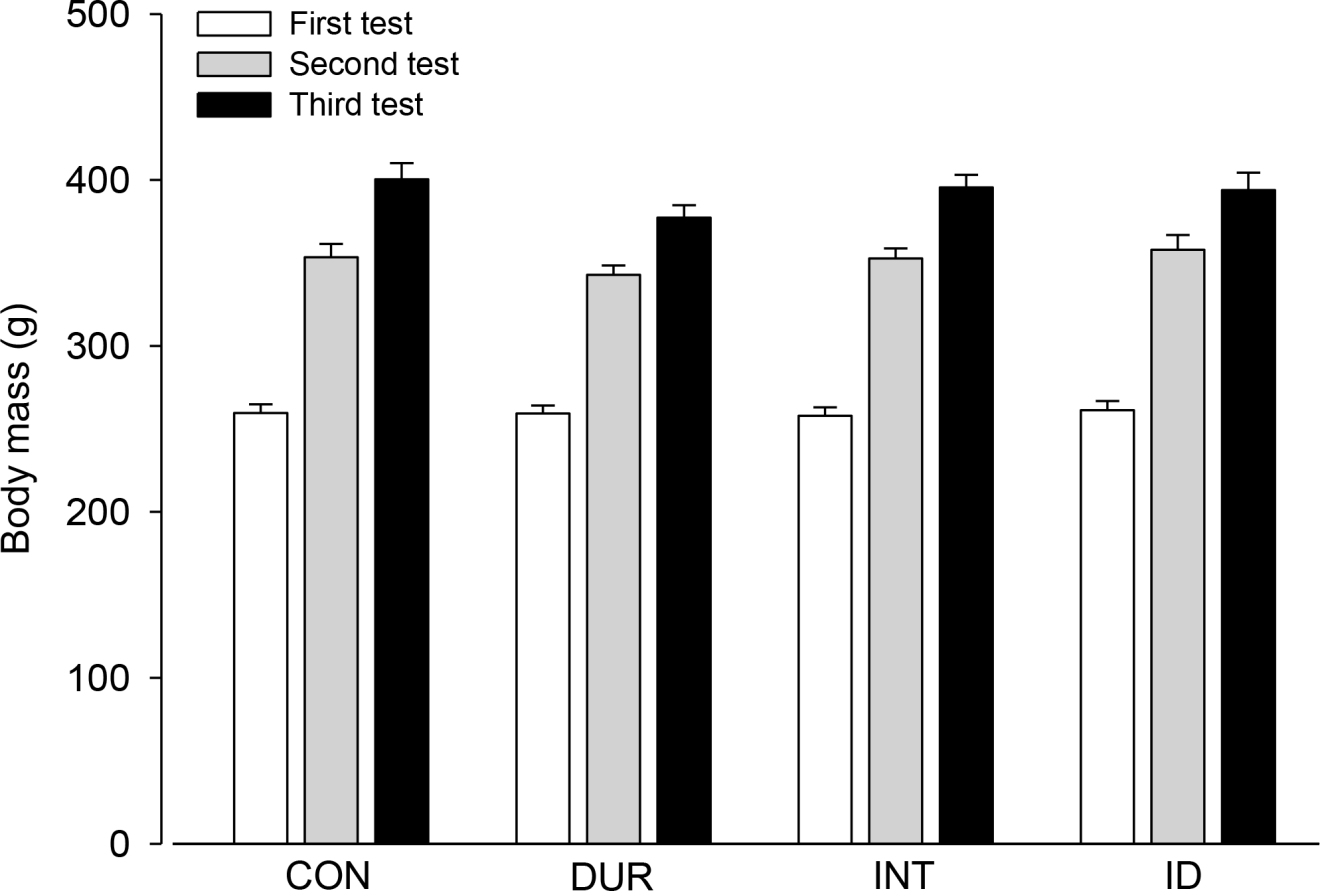
Body mass of the rats. Body mass was measured at three different time points: incremental exercises (tests) performed prior to the training (or control) protocols and at the end of the 4^th^ and 8^th^ weeks. Two-way ANOVA yielded the following results: group effect (F = 0.754, *p* = 0.523), moment effect (F = 833.941, *p* < 0.001) and interaction between the two factors (F = 1.299, *p* = 0.261). This analysis indicated that, irrespective of the group, body mass was higher in the second than in the first test and higher in the third than in the second and first tests.

During the 8-week period, the rats in all the experimental groups exhibited progressive increases in body mass, with a final average body mass of approximately 400 g for all groups. Notably, aerobic training and the training load components, i.e., intensity and duration, did not influence body mass gain (Fig 3).

**Fig 4:**
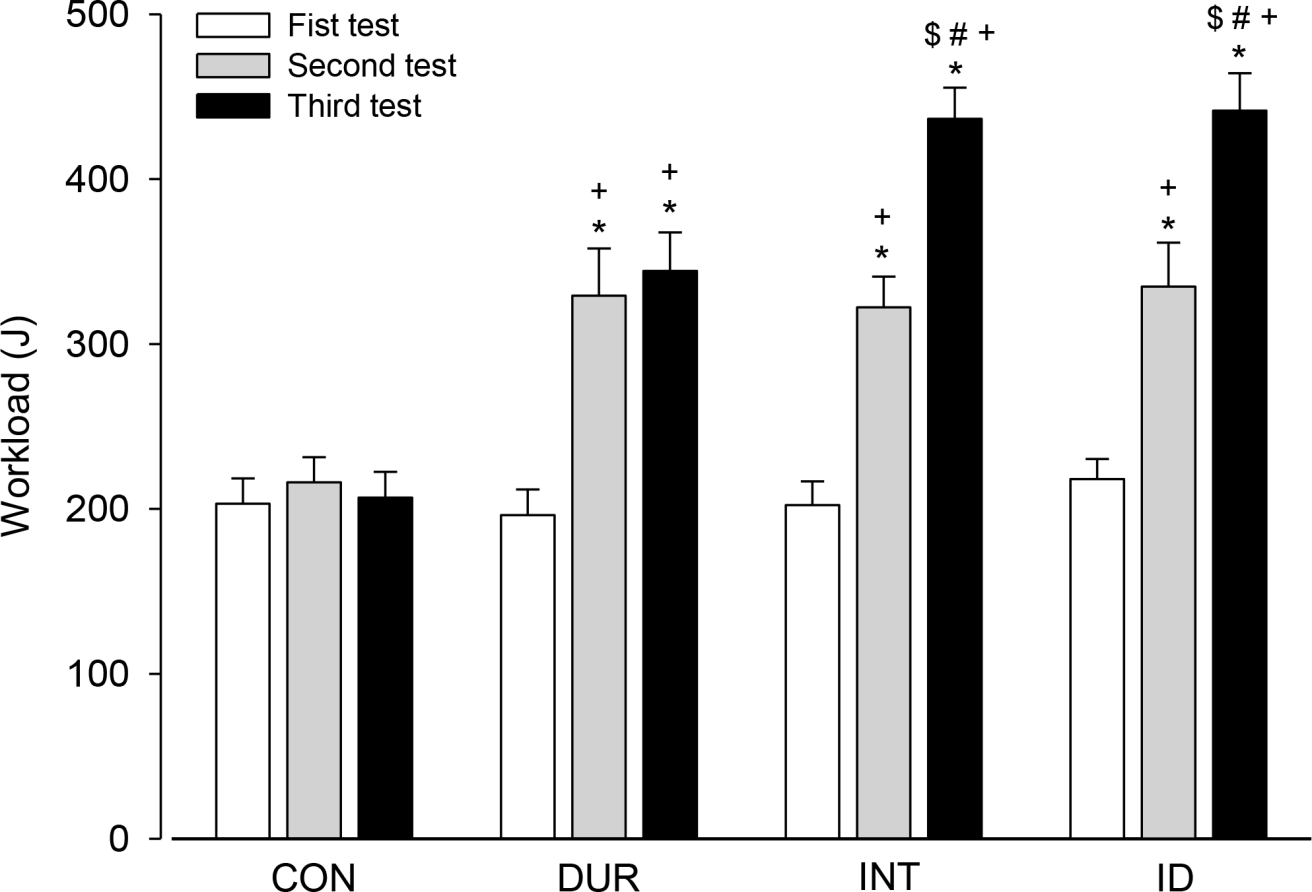
Physical performance of the rats. Physical performance was measured at three different time points: incremental exercises (tests) performed prior to the training (or control) protocols and at the end of the 4^th^ and 8^th^ weeks. Performance was evaluated by calculating the workload. Two-way ANOVA yielded the following results: group effect (F = 13.583, *p* < 0.001), moment effect (F = 100.080, *p* < 0.001) and interaction between the two factors (F = 12.906, *p* < 0.001). * Significantly different relative to the first test in the same group (*p* < 0.05). + Significantly different relative to the CON group in the same test (*p* < 0.05). # Significantly different relative to the second test in the same group (*p* < 0.05). $ Significantly different relative to the DUR group in the same test (*p* < 0.05; n = 21 rats per experimental group).

We subsequently assessed the rats’ performance by calculating their workload, a performance index that considers body mass (Fig 4). The CON group performed a similar workload in the three tests during the training protocol (first = 203.1 ± 15.5 J; second = 216.1 ± 15.3 J; and third = 206.8 ± 15.6 J). All trained groups exhibited an increased workload in the second test compared with those in the first test (DUR: 329.3 ± 28.8 vs. 196.3 ± 15.5 J, respectively; INT: = 322.2 ± 18.6 vs. 202.3 ± 14.4 J, respectively; and ID: 334.8 ± 26.6 vs. 218.1 ± 12.2 J, respectively). The trained groups also exhibited an increased workload compared with the CON group in the second (CON = 216.1 ± 15.3 J vs. DUR = 329.3 ± 28.8 J vs. INT = 322.2 ± 18.6 J vs. ID = 334.8 ± 26.6 J) and third tests (CON = 206.8 ± 15.6 J vs. DUR = 344.3 ± 23.3 J vs. INT = 463.6 ± 18.8 J vs. ID = 441.5 ± 22.7 J). In addition, the INT and ID groups but not the DUR group performed higher workloads in the third test than in the second test (INT: 436.6 ± 18.8 vs. 322.2 ± 18.6 J, respectively; ID: 441.5 ± 22.8 vs. 334.8 ± 26.6 J, respectively). Therefore, the workload performed by the INT and ID rats in the third test was increased compared with the performance by the DUR rats (Fig 4).

**Fig 5:**
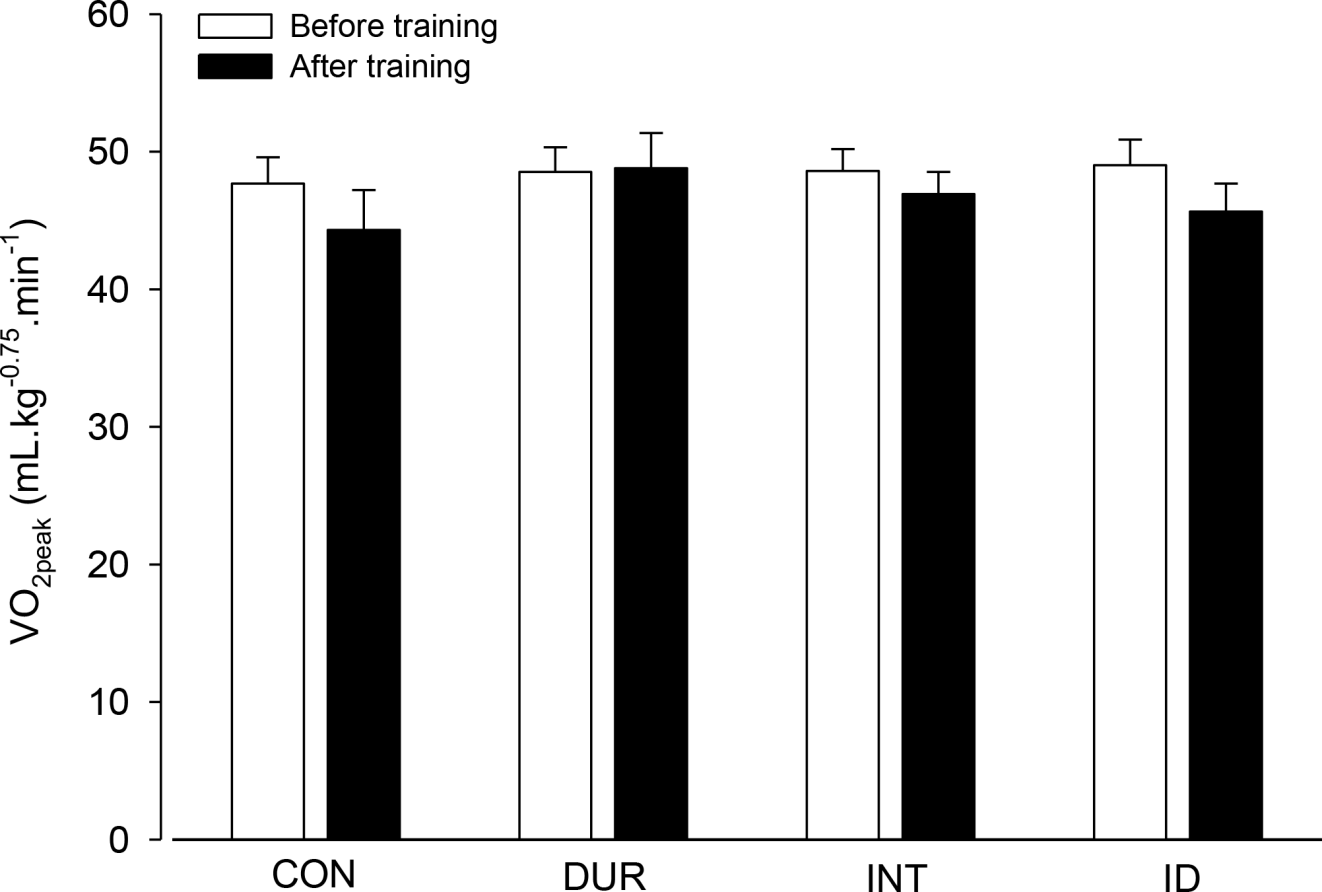
Peak rate of oxygen consumption (VO_2peak_) of the rats. VO_2peak_, adjusted for the rat’s size (kg^-0.75^), was measured at two different time points: incremental exercises performed before and after the training (or control) protocols. Two-way ANOVA yielded the following results: group effect (F = 0.696, *p* = 0.562), moment effect (F = 1.589, *p* = 0.218) and interaction between the two factors (F = 0.287, *p* = 0.835; n = 9 rats for the CON group and n = 8 rats for the other groups).

**Fig 6:**
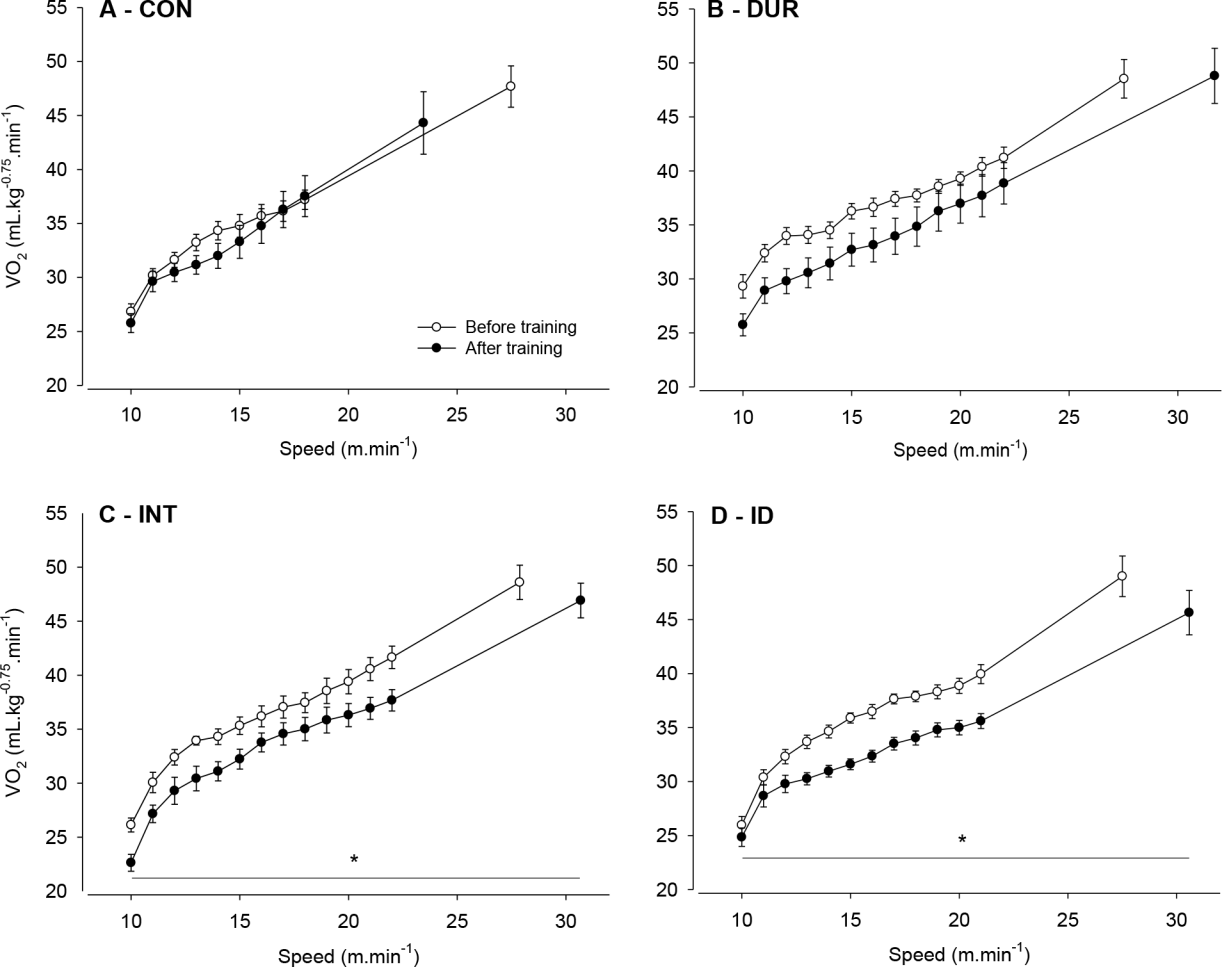
VO_2_ (mL.kg^-0.75^.min^-1^) of the rats during the incremental exercises. Incremental exercises were performed at two different time points: before and after the training or control protocols in the CON (panel A), DUR (panel B), INT (panel C) and ID (panel D) groups. Two-way ANOVA yielded the following results: moment effect (A: F = 0.720, *p* = 0.409; B: F = 3.697, *p* = 0.075; C: F = 7.343, *p* = 0.017; D: F = 23.762, *p* < 0.001), running speed effect (A: F = 85.726, *p* < 0.001; B: F = 69.683, *p* < 0.001; C: F = 128.777, *p* < 0.001; D: F = 104.292, *p* < 0.001) and interaction between the two factors (A: F = 0.806, *p* = 0.611; B: F = 0.782, *p* = 0.678; C: F = 0.378, *p* = 0.975; D: F = 0.994, *p* = 0.457). * Significant major effect of moment (*p* < 0.05). Differences in VO_2_ caused by running speeds within the experimental groups are not displayed in the figure to avoid an excess description of obvious information; n = 9 rats for the CON group and n = 8 rats for the other groups.

We then assessed the rats’ VO_2peak_ adjusted to their size, which was measured before and after the 8-week protocol. Before the control or training protocols, VO_2peak_ values were positively correlated with workload performed by the rats (r = 0.691; *p* < 0.001). Unexpectedly, no training effects and no intergroup differences were identified in this physiological parameter (Fig 5). Following the 8-week period, VO_2peak_ values were still positively correlated with workload (r = 0.424; *p* < 0.05), but the Pearson’s correlation coefficient was lower than that observed at the beginning of the experiment. Although no differences were identified in VO_2peak_, the VO_2_ at submaximal intensities of incremental exercise was lower in the INT and ID groups after aerobic training than prior to aerobic training (Fig 6C and 6D). This response was not evidenced in the CON (Fig 6A) or DUR (Fig 6B) groups.

**Fig 7:**
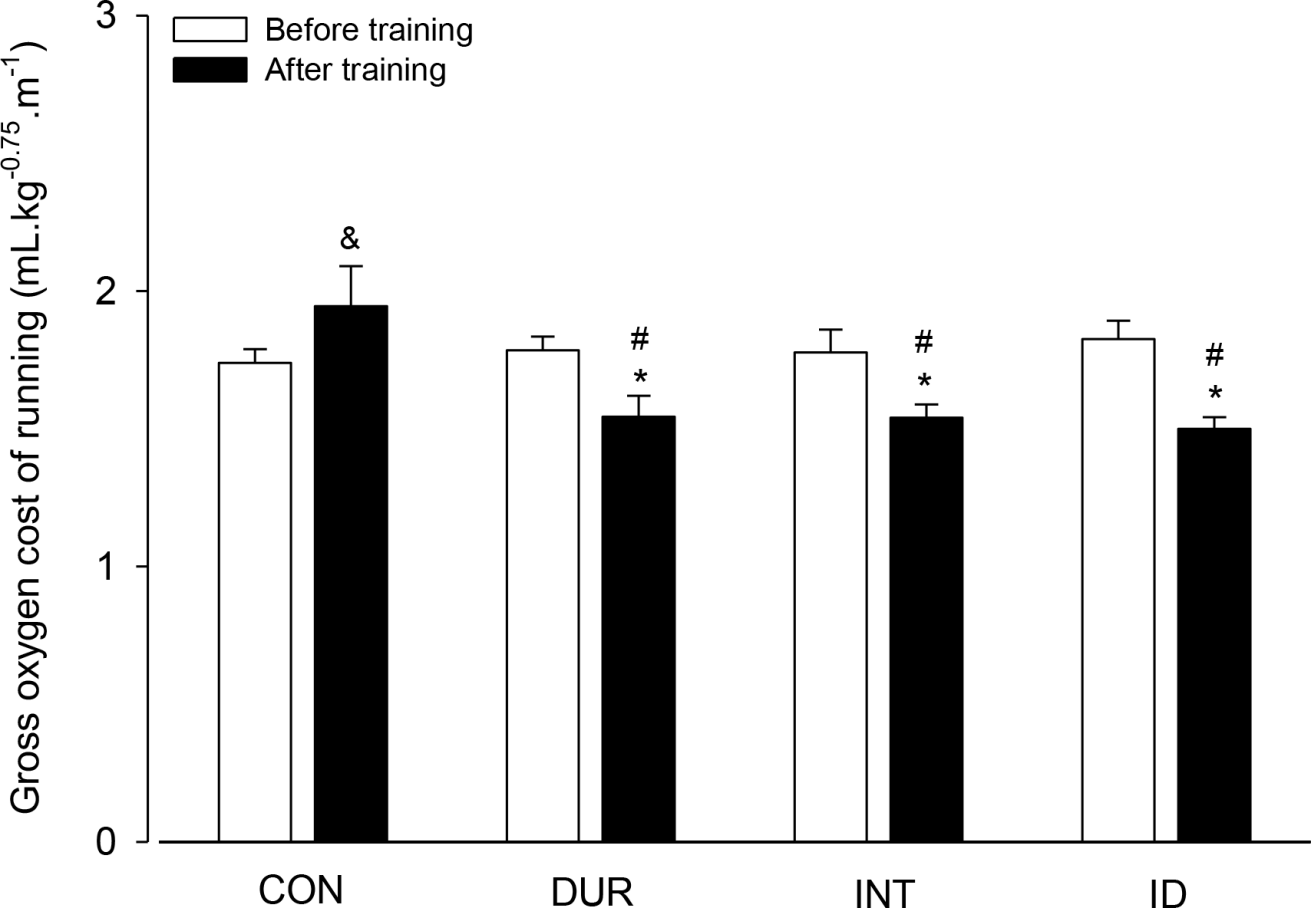
Gross oxygen cost of running (mL.kg^-0.75^.m^-1^) of the rats. The gross oxygen cost of running was calculated at the last completed stage during incremental exercises performed before and after the training (or control) protocols. Two-way ANOVA yielded the following results: group effect (F = 2.424, *p* = 0.086), moment effect (F = 7.968, *p* = 0.009) and interaction between the two factors (F = 5.450, *p* = 0.004). * Significantly different relative to the measurement obtained prior to the training protocols (*p* < 0.05). # Significantly different relative to the CON group in the same test (*p* < 0.01). & Trend toward a significant difference relative to the measurement obtained prior to the control protocol (*p* = 0.051; n = 9 rats for the CON group and n = 8 rats for the other groups).

The finding that all trained groups reached similar VO_2peak_ values at higher speeds after the training protocols suggested a training-induced reduction in the gross oxygen cost of running, which corresponds to an improvement in running economy. Indeed, the running economy of the trained rats at the last completed stage was increased after the training protocols compared with that prior to the training protocols, whereas the CON rats tended to display reduced economy (*p* = 0.051). Furthermore, all trained groups exhibited increased running economy compared with the CON group at the end of the 8 weeks (Fig 7).

**Fig 8:**
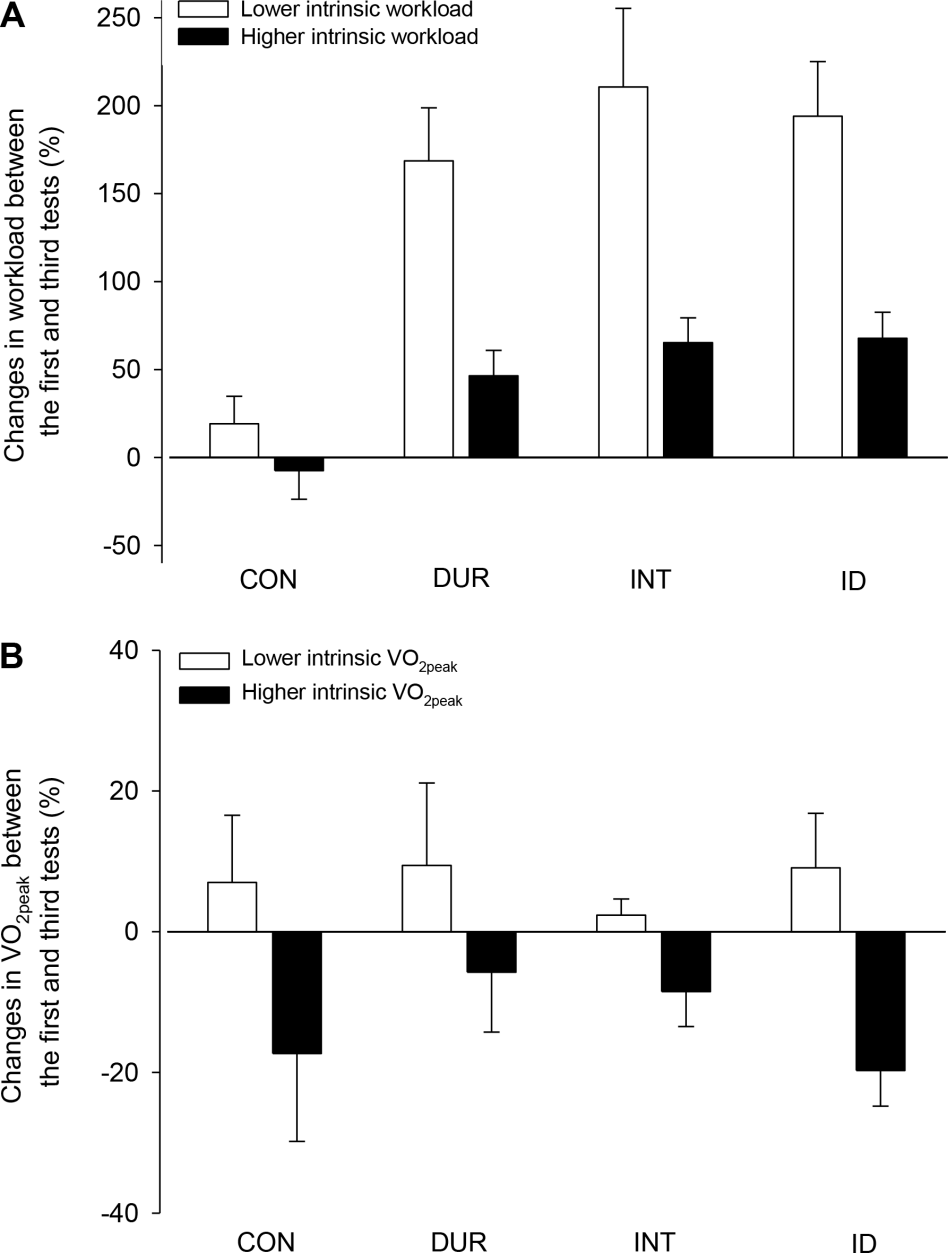
Changes in performance between the first and third tests in the four experimental groups. Each group was subdivided into rats with lower and higher intrinsic aerobic capacities, as determined by a performance evaluation in the first incremental exercise. Two-way ANOVA of the data presented in panel A (workload) yielded the following results: group effect (F = 11.980, *p* < 0.001), aerobic capacity effect (F = 35.297, *p* < 0.001) and interaction between the two factors (F = 2.268, *p* = 0.092). Two-way ANOVA of the data presented in panel B (VO_2peak_) yielded the following results: group effect (F = 0.311, *p* = 0.817), aerobic capacity effect (F = 10.936, *p* = 0.003) and interaction between the two factors (F = 0.473, *p* = 0.704; n = 9 rats for the control group and n = 8 rats per training group, with 4 rats with a higher initial VO_2peak_ and 4 rats with a lower initial VO_2peak_ being used in this analysis).

The final analyses consisted of subdividing the rats from each group into groups with lower and higher intrinsic aerobic capacities (either workload or VO_2peak_), as assessed in the first incremental test. Regarding the workload (Fig 8A), each group (n = 21) was subdivided into groups containing 7 rats with higher initial workloads and 7 rats with lower initial workloads; the 7 rats with intermediate workloads were excluded from this analysis. As expected, the trained rats, irrespective of the lower or higher initial workloads, exhibited greater improvements than the CON rats (Fig 8A). In addition, irrespective of the group (i.e., CON, DUR, INT or ID), the rats with higher initial workloads exhibited smaller improvements than the rats with lower initial workloads.

Regarding the VO_2peak_ (Fig 8B), each group was subdivided into groups containing 4 rats with a higher initial VO_2peak_ and 4 rats with a lower initial VO_2peak_; in the CON group (n = 9), the rat with the intermediate VO_2peak_ was excluded from this analysis. No intergroup differences were identified in the VO_2peak_ changes between the third and first tests. In contrast, an intrinsic capacity-associated effect was identified. In the four experimental groups, the rats with a lower initial VO_2peak_ exhibited positive changes in this measure across the 8 weeks, whereas the rats with a higher initial VO_2peak_ exhibited negative changes (Fig 8B).

## Discussion

The current findings show that the three aerobic training protocols improved the physical performance of the rats subjected to incremental exercises (Fig 4). Nevertheless, the performance gain reached a plateau earlier in the group with a predominant overload in duration than in the other training groups. Thus, at the end of training, the performance gain identified in the DUR group was lower than that in the groups with a marked overload in intensity (i.e., the INT and ID groups; Fig 4A and 4B). This finding confirmed our hypothesis that a predominant overload in the intensity of aerobic training sessions would produce a greater improvement in physical performance. Furthermore, none of the training protocols changed the VO_2peak_ values; however, these values were attained at faster speeds, which indicated increased running economy. The lack of improvement in VO_2peak_ and of different gains in running economy induced by the three training protocols refuted our hypotheses.

The selection of a specific %VO_2max_ for training sessions and the maintenance of this intensity across a training protocol is a common procedure adopted in rat studies [13, 14, 25]. The maintenance of a specific %VO_2max_ is reached by regulating the treadmill speed (most common) or inclination, which thus ensures the individualization of the training overload. Because our aim was to investigate the effect of predominant overload on the intensity or duration of sessions and because the total distance covered among the three groups was matched in every training session, we could not ensure overload individualization. However, the application of overload was evident in the three training groups, as evidenced by increases in running duration and speed (Fig 1A and 1B). Not only the absolute speed but also the speed relative to the S_max_ was increased during the training protocols; in fact, the speed in the last training session corresponded to a higher %S_max_ relative to the speed in the first session in the trained groups (Fig 2A). As expected, the overload in intensity was higher, intermediate and lower in the INT, ID and DUR groups, respectively. In contrast, the overload in duration was higher, intermediate and lower in the DUR, ID and INT groups, respectively.

To minimize the effect of body mass gain on physical performance, the total workload performed by the rats during the incremental exercises was calculated. The workload was similar for the CON group over the 8 weeks (Fig 4); these data indicate that 40 sessions with a 2-min duration do not provide physiological stimuli that generate the adaptations responsible for improved performance. In contrast, the trained groups exhibited training-mediated increases in workload. Interestingly, the DUR group reached a plateau in workload from the 4^th^ week of training, and the INT and ID rats exhibited higher workloads than the DUR group at the end of training. These intergroup differences in the kinetics of performance gains can be explained by differences in the intensity of training sessions.

In the last session, the DUR group ran at an intensity that corresponded to 58% of the VO_2peak_ (Fig 2B), which represents moderate-intensity exercise according to guidelines for prescribing exercise for humans [26]. In addition, the DUR rats completed the training protocol running at 43% of S_max_, an intensity that is below the lactate threshold for untrained rats, which has been reported at different intensities, including running at approximately 50% [27], 57% [28] or 75% of S_max_ [29]. Thus, this moderate exercise performed by the DUR rats likely limited additional performance gains as the training protocol continued, despite the marked increases in the sessions’ duration and training volume (Fig 1B). Because the INT and ID rats completed the last training session running at intensities that corresponded to 89% and 75% of the VO_2peak_ (Fig 4B), respectively, it is possible that higher intensities (which are classified as vigorous strain [26]) are required to induce the profound adaptations that underlie sustained improvements in performance. Notably, the speed at which the INT and ID rats ran during the last training session was above the lactate threshold for untrained rats, according to more conservative estimates [27, 28]. Interestingly, the ID group exhibited a performance similar to the INT group (Fig 4), suggesting that within the time frame investigated, effective aerobic training requires an overload in intensity; however, it does not need to be exclusive and may be associated with an overload in duration in an alternating manner.

The initial fitness level may modulate training responses in humans [2, 30]. Indeed, a study conducted with 633 individuals subjected to a 20-week training program showed that baseline VO_2max_ values correlated significantly, but weakly (r = -0.37; *p* < 0.001), with training-induced percent changes in the VO_2max_ [31]. In the present study, the rats with lower intrinsic endurance exhibited increased performance gains (workload improvement) after being subjected to the three training protocols (Fig 8A). Along the same lines, the rats with lower intrinsic VO_2peak_ values exhibited positive changes in this physiological parameter following aerobic training, whereas the rats with higher VO_2peak_ values exhibited negative changes. Interestingly, the predominant overload in the intensity or duration of sessions did not differentially modulate the changes in workload or VO_2peak_ in the rats with lower and higher initial levels of fitness (Fig 8A and 8B). These findings indicate that among the currently assessed training protocols, no particularly effective protocol for developing aerobic capacity in rats with different intrinsic capacities was identified.

The current training protocols failed to improve the VO_2peak_ in rats (Fig 5), which disagrees with previous findings [13, 25]. These contradictory reports may be explained by the different exercise intensities employed during the training sessions. Whereas Wisloff et al. [25] and Kemi et al. [13] subjected rats to high- (85–90% of VO_2max_) or moderate-intensity (65–70% of VO_2max_) interval trainings, it is likely that our rats achieved these moderate-to-high intensities only during the last weeks of training. Another reasonable explanation for the absence of improvement in the VO_2peak_ after training is the expected age-related reduction in this physiological parameter. Because our rats initiated the familiarization protocol at 12 weeks of age, they concluded training at 21 weeks of age, which represents an age at which a decrease in VO_2max_ is expected, according to Pica and Brooks [32]. These authors showed that the absolute and relative VO_2max_ increased in untrained and trained female rats until 20 weeks of age. The adult untrained rats subsequently exhibited a reduction in VO_2max_, whereas the trained rats maintained the level of this physiological parameter, although a statistical trend toward a VO_2max_ reduction was identified. However, the latter explanation may not be the best; Wisloff et al. [25] and Kemi et al. [13] initiated their protocols using rats of the same age and body mass as those used in the present study.

Another possible explanation for the lack of improvement in VO_2peak_ after training is the exercise protocol used for VO_2_ testing. We conducted prolonged incremental-speed running because long-lasting efforts were associated with greater VO_2max_ [15], a finding that was reproduced in the present study. Nevertheless, Wisloff et al. [25] showed that the treadmill inclination significantly affected the highest oxygen uptake measured, with VO_2peak_ values being greater at an inclination of 25° than at 0, 15, 20 or 30°. The use of 5° inclination in the present study intended to standardize inclination during training sessions and tests for determining performance but may have hindered observations of training-induced adaptations if the true VO_2max_ was not reached by the rats [25].

In humans, VO_2max_ (or VO_2peak_) is not the only physiological parameter that modulates aerobic performance [9]. In agreement with this finding, a dissociation between improvements in performance and VO_2max_ has also been observed in rats; enhanced performance (time to exhaustion) was exhibited at the 4^th^ week after training was initiated, whereas the VO_2max_ was increased only at the 12^th^ week [33]. In the present study, the training-induced adaptations enabled the rats to attain increased treadmill speeds, despite the finding that their VO_2peak_ values were similar to those recorded prior to the training protocol (Fig 6). The latter result suggests that the three training protocols improved the running economy of the rats, which, in fact, did occur (Fig 7). Interestingly, Wisloff et al. [25] suggested that training at lower intensities may substantially improve running (work) economy without altering the VO_2max_; moreover, increased economy may imply increased work capacity and could therefore be a more relevant measure to assess the effect of specific training regimens, which appeared to be the case in the present study.

The observed changes in running economy may be explained by biomechanical factors, including improved technique and the transfer of elastic energy during stretch-shortening cycles [34], as well as by physiological adaptations in skeletal muscle, including an increase in mitochondrial content, which results in increased respiratory capacity [35, 36]. Whereas these biomechanical factors have never been studied in rats, aerobic training induces several skeletal muscle adaptations in this species [37, 38]. A consequence of these mitochondrial adaptations is the increased utilization of fat during submaximal exercise, with a proportional decrease in carbohydrate utilization [39]. Collectively, these adaptations may contribute to increased endurance and a raised lactate threshold in the trained state, i.e., to exercise at a higher %VO_2max_ [39]. Muscular adaptations in response to aerobic training have also been associated with VO_2max_ improvements [40].

The greater gains in performance exhibited by the INT and ID groups compared with those of the DUR group cannot be explained by different changes in the VO_2peak_ (Fig 5) or running economy (Fig 7). Aside from the VO_2max_ and running economy, the ergogenic effects of aerobic training may be explained by changes in the lactate threshold [41]. Therefore, the different gains in performance between our trained groups are likely explained by differences in the speed at which the lactate threshold was reached. A recent comparison between two training protocols (longer-duration and low-intensity vs. high-intensity interval training) showed that the lactate threshold was reached at a higher power output in trained rowers subjected to high-intensity training [42]. Similar evidence has not been provided for laboratory rats; de Araujo et al. [43] compared the effects of two training protocols (continuous vs. interval) on rats’ lactate threshold, but neither protocol was effective at increasing the running speed associated with the lactate threshold.

In conclusion, the three studied aerobic training protocols improved the physical performance of rats subjected to incremental-speed treadmill running. The group with a predominant overload in duration exhibited a plateau in performance during the last 4 weeks of the study, whereas the groups with an overload in intensity did not exhibit this plateau. This observation resulted in an increased performance of the INT and ID groups compared with that in the DUR group at the end of the training protocol. In addition, the evaluated training protocols did not induce changes in VO_2peak_, despite the observation that this physiological parameter was reached at higher speeds, indicating increased running economy after training. Finally, rats with lower intrinsic endurance displayed greater improvements in performance induced by the three evaluated trainings than rats with higher intrinsic endurance.

## Supporting information

S1 Table Sessions of aerobic training with predominant overload in duration. (DOCX)

S2 Table Sessions of aerobic training with predominant overload in intensity. (DOCX)

S3 Table Sessions of aerobic training with alternating and similar overloads in intensity and duration. (DOCX)
