# Supplementary material for: Effects of manipulating the duration and intensity of aerobic training sessions on the physical performance of rats
Source: PLoS One. 2017 Aug 25;12(8):e0183763. doi: 10.1371/journal.pone.0183763 (PMC5571967; doi:10.1371/journal.pone.0183763)
Supplement: S1 Table — (DOCX) [file pone.0183763.s001.docx]

**S1 Table.** Sessions of aerobic training with predominant overload in duration.

| Week | 1^st^ day  (min – m/min) | 2^nd^ day  (min – m/min) | 3^rd^ day  (min – m/min) | 4^th^ day  (min – m/min) | 5^th^ day  (min – m/min) |
| --- | --- | --- | --- | --- | --- |
| 1^st^ | 30.0 – 8.0 | 30.0 – 8.0 | 30.0 – 8.0 | 30.0 – 8.0 | 37.5 – 8.0 |
| 2^nd^ | 37.5 – 8.0 | 37.5 – 8.0 | 37.5 – 8.0 | 45.0 – 8.3 | 45.0 – 8.3 |
| 3^rd^ | 45.0 – 8.3 | 45.0 – 8.3 | 52.5 – 8.6 | 52.5 – 8.6 | 52.5 – 8.6 |
| 4^th^ | 52.5 – 8.6 | 60.0 – 9.0 | 60.0 – 9.0 | 60.0 – 9.0 | 60.0 – 9.0 |
| 5^th^ | 67.5 – 9.3 | 67.5 – 9.3 | 67.5 – 9.3 | 67.5 – 9.3 | 75.0 – 9.8 |
| 6^th^ | 75.0 – 9.8 | 75.0 – 9.8 | 75.0 – 9.8 | 82.5 – 10.2 | 82.5 – 10.2 |
| 7^th^ | 82.5 – 10.2 | 82.5 – 10.2 | 90.0 – 10.7 | 90.0 – 10.7 | 90.0 – 10.7 |
| 8^th^ | 90.0 – 10.7 | 97.5 – 11.1 | 97.5 – 11.1 | 97.5 – 11.1 | 97.5 – 11.1 |
