# Supplementary material for: Effects of manipulating the duration and intensity of aerobic training sessions on the physical performance of rats
Source: PLoS One. 2017 Aug 25;12(8):e0183763. doi: 10.1371/journal.pone.0183763 (PMC5571967; doi:10.1371/journal.pone.0183763)
Supplement: S2 Table — (DOCX) [file pone.0183763.s002.docx]

**S2 Table.** Sessions of aerobic training with predominant overload in intensity.

| Week | 1^st^ day  (min – m/min) | 2^nd^ day  (min – m/min) | 3^rd^ day  (min – m/min) | 4^th^ day  (min – m/min) | 5^th^ day  (min – m/min) |
| --- | --- | --- | --- | --- | --- |
| 1^st^ | 30.0 – 8 | 30.0 – 8 | 30.0 – 8 | 30.0 – 8 | 30.0 – 10 |
| 2^nd^ | 30.0 – 10 | 30.0 – 10 | 30.0 – 10 | 31.3 – 12 | 31.3 – 12 |
| 3^rd^ | 31.3 – 12 | 31.3 – 12 | 32.1 – 14 | 32.1 – 14 | 32.1 – 14 |
| 4^th^ | 32.1 – 14 | 33.8 – 16 | 33.8 – 16 | 33.8 – 16 | 33.8 – 16 |
| 5^th^ | 35.0 – 18 | 35.0 – 18 | 35.0 – 18 | 35.0 – 18 | 36.8 – 20 |
| 6^th^ | 36.8 – 20 | 36.8 – 20 | 36.8 – 20 | 38.2 – 22 | 38.2 – 22 |
| 7^th^ | 38.2 – 22 | 38.2 – 22 | 40.0 – 24 | 40.0 – 24 | 40.0 – 24 |
| 8^th^ | 40.0 – 24 | 41.5 – 26 | 41.5 – 26 | 41.5 – 26 | 41.5 – 26 |
